# Understanding the occupational identity of care-givers for people with mental health problems

**DOI:** 10.1177/03080226211018153

**Published:** 2021-05-18

**Authors:** Megan L Howes, Diane Ellison

**Affiliations:** Occupational Therapy Department, 98967 Brunel University, Uxbridge, UK

**Keywords:** Mental health care-givers, occupational identity theory, occupational therapy, occupational participation

## Abstract

**Introduction:**

There is recognition within the literature that the role of care-giving can have a negative impact on care-givers’ general well-being. Less is understood about the role of care-giving on an individual’s occupational participation and in turn occupational identity. Occupational therapists have a unique understanding of the interplay between occupational participation and health, though this is an area that has been under researched in relation to mental health care-givers. Therefore, the current research aims to understand how the role of care-giving for an individual with a mental illness impacts on occupational participation and identity.

**Method:**

A qualitative semi-structured interview the Occupational Performance and History Interview–Version 2 was utilised to understand life experiences. Six mental health care-givers were interviewed, and these interviews were transcribed for thematic analysis.

**Findings:**

Three main themes were identified: being me, roles and responsibilities associated with care-giving and services.

**Conclusion:**

The findings suggest being a mental health care-giver does have a detrimental impact on occupational participation and therefore occupational identity. As care-givers gained more experience in their role, they used occupational adaption as a positive coping mechanism that helped them achieve occupational balance. Using their unique understanding of occupational participation and occupational identity, occupational therapists are well placed to utilise their knowledge and skills to work in a systemic way supporting both the person with mental illness and their care-giver.

## Introduction

A care-giver is defined as ‘an unpaid individual involved in assisting others with activities of daily living and/or medical tasks’ (Family Caregiver Alliance, 2019). The World Health Organization (WHO) (2015) estimate there are 349 million care-dependent people worldwide. There are no available statistics for worldwide care-givers. In the 2011 United Kingdom (UK) census, there were approximately 5.8 million carers; this figure is said to be rising faster than the pace of the population (Office for National Statistics, 2013). Using these statistics, combined with their own surveys, Carers UK (2019) estimate there were 8.8 million care-givers in 2019. Care-givers are estimated to save UK health and social care services £132 billion per year (Carers UK, 2018).

There is recognition among the literature that care-giving can cause a substantial burden for individuals, impacting on their general well-being. This is particularly problematic for mental health care-givers due to the unpredictable and non-reciprocal nature of caring (Lawn and McMahon, 2014; Weimand et al., 2013). When researching mental health care-givers in the UK, Rowe (2012) found this population felt unprepared for the role with a sense of obligation placed on them by both themselves and professionals.

Occupational therapists in Australia (Bourke et al., 2015) offer an occupational understanding to mental health care-giver roles and provide interventions to support with their role. However, due to contextual and service differences it is unclear as to whether this is transferable to the UK. Occupational therapy research in the UK focuses on care-givers of those with dementia or physical illness; none were found for mental health care-givers. Given their unique understanding of the interplay between occupation and health, occupational therapists may offer a new perspective on mental health care-givers within a UK context. Therefore, this study aims to understand the lived experience of mental health care-givers in the UK, specifically how being a mental health care-giver impacts on occupational identity.

### Occupational identity

**Figure 1.**
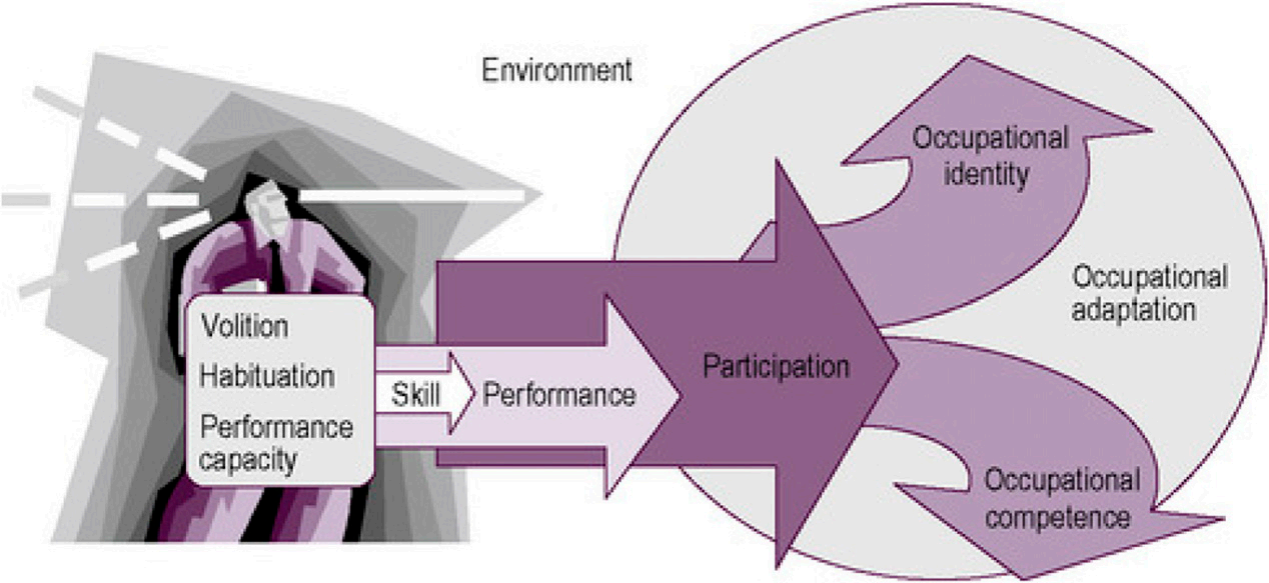
The process of occupational adaptation (Taylor, 2017).

Occupational identity was a term initially coined by Christiansen (1999) as the way in which individuals display their personal identity. Occupational identity can be defined as ‘a composite sense of who one is and wishes to become as an occupational being generated from one’s history of occupational participation, volition, habituation, and experience as a lived body’ (Kielhofner, 2008a, p. 106). Occupational identity and occupational competence are seen as intertwined entities that co-develop over time. Occupational competence is defined by Kielhofner (2008, p. 106) as ‘the degree to which one sustains a pattern of occupational participation that reflects one’s occupational identity’. Occupational competence and occupational identity are formed by occupational participation, as can be seen in the process of occupational adaptation (Taylor, 2017; Figure 1).

When individuals experience threats to occupational competence and their occupational identity, occupational adaptation occurs (Taylor, 2017; see Figure 1). Researchers found families experienced threats to participation in leisure and social activities and adapted their occupational participation in order to meet the demands of their caring role (Acero et al., 2017; Weimand et al., 2013). Occupational therapists currently use their knowledge of occupational adaptation to support those with mental illness; however, less is understood about how this relates to mental health care-givers.

Phelan and Kinsella (2009) identified four key assumptions at the core of the occupational identity theory: (1) individuals are at the core of identity formation, (2) individuals have choice, (3) occupations offer productivity and (4) social dimensions shape occupational identity. It appears important to critically consider these assumptions in relation to care-givers, whilst recognising the Western orientation of this theory.

1. Individuals are at the core of identity formation

The first assumption offers some potential difficulties for the care-giving population, as the nature of care-giving is not individualistic, instead families were found to grow as a group (Acero et al., 2017). Research into mental health care-givers recognised cultural differences, with those in Africa having a more family-orientated identity and those in Western countries experiencing more individualistic identity with an emphasis on independence for individuals (Acero et al., 2017; Marimbe et al., 2016). It is unclear how these differences impact on the care-givers’ experience, though cultural values may impact on the formation of occupational identity.

2. Individuals have choice

There is a lack of understanding among the occupational identity theory regarding occupations arising out of duty or obligation (Phelan and Kinsella, 2009), which appears to often be the case with care-givers who engage in this occupation due to a sense of love, responsibility and values system (Lawn and McMahon, 2014; McAuliffe et al., 2014; Weimand et al., 2013). Acero et al. (2017) recognised cultural differences in studies they reviewed with those in Taiwan and in the UK of Pakistani origin, experiencing less choice in their caring role. Cultural values meant there was an expectation that they played an active part in their relative’s care. This expectation was less evident in those from Western cultures.

3. Occupations offer productivity

The role of care-giving can offer a sense of productivity although it can also reduce productivity in other roles. When researching care-givers of those with Alzheimer’s disease, Pearlin et al. (1990) introduced the idea of care-givers feeling a sense of captivity. They described this as a care-giver being compelled to engage in the role and there being a reduction in choice of activities. This could lead to care-givers experiencing a loss of identity.

When looking specifically at mental health care-givers, findings suggest an ability to participate in other productive roles is impacted. Some gave up work, changed their role at work or reduced working hours to meet caring demands (Ae-Ngibise et al., 2015; Gater et al., 2014). Occupational therapists can support care-givers to maintain productive and meaningful activity independent of their care-giving role (Chaffey and Fossey, 2004).

Differences in care provision across countries may impact on care-givers productivity. WHO (2011) highlights the difference and inadequacies in mental health care in low-to-middle-income countries compared to high-income countries, where community-based resources are more available. Having resources available for those in high-income countries is likely to allow care-givers more occupational balance compared to those in low-to-middle-income countries, which may impact on engagement in productive and leisure activities.

4. Social dimensions shape occupational identity

Phelan and Kinsella (2009) suggest social approval may form, shape and even produce occupational identity. Research suggests care-givers adapted their social behaviour due to the care-recipients’ illness; individuals reported fewer social opportunities and forming of relationships (Acero et al., 2017). Mental health care-givers reported being blamed by the local community for their family members’ illness, due to Eastern cultural beliefs that mental illness is the result of parents’ sins (Marimbe et al., 2016). Similar experiences were found in Western cultures with parents of adult-children with severe mental illness feeling blamed, rejected and devalued by friends, neighbours, relatives and professionals (Weimand et al., 2013; Weiss et al., 2013).

Social growth was found to be stunted in this population due to stigma and lack of social support, therefore impacting on both social and occupational identity (Acero et al., 2017; Marimbe et al., 2016; Weiss et al., 2013).

Given the familial-orientated nature of care-giving, more information is required to understand how occupational identity is experienced by care-givers. This research study aims to understand the experiences of mental health care-givers and answer the question: does caring for a person with a mental illness impact on care-givers occupational identity?

## Method

### Design

A qualitative semi-structured interview was utilised in order to understand life experiences using the Occupational Performance and History Interview–Version 2 (OPHI-II; Kielhofner et al., 2004). The OPHI-II interview guide includes questions to facilitate the gathering of information about the individual’s occupational choices and roles, daily routines, critical life events and environmental influences on his/her occupations (Kielhofner et al., 2004). The OPHI-II consists of three parts: (a) the semi-structured interview explores occupational life history; (b) a rating scale offers a measure of the individual’s occupational identity, occupational competence and the impact of the environment; and (c) a life history narrative designed to capture qualitative features of the life history. Part A of the OPHI-II was utilised for this dissertation. It was decided Parts B and C would not be utilised. The quantitative measure in Part B would not offer much knowledge to the research due to the small number of participants, and Part C compromised anonymity of participants. Part A is deemed a reliable and valid assessment tool when used alone (Kielhofner et al., 2004).

Previous studies suggest OPHI-II is valid, reliable and a useful tool for exploring the occupational lives of people with medical conditions (Gray and Fossey, 2003; Kielhofner et al., 2004). Whilst the current research is not concerned primarily with participants’ medical conditions, the OPHI-II has been used in research with the care-giving population, with positive results offering an understanding of the life transitions and occupational issues faced by mental health care-givers (Chaffey and Fossey, 2004).

Six adults who identified as care-givers for a person with a mental illness were interviewed. Data were analysed using thematic analysis. This was preferred due to its relative flexibility and opportunity for the research question to evolve throughout the coding and theme development process (Braun and Clarke, 2006).

Ethical application was attained through Brunel University’s Research Ethical Committee.

### Sampling strategy

A purposive sampling strategy was utilised, with inclusion criteria being participants identified as a care-giver of someone with a mental health diagnosis (not including dementia, learning disability or neurological conditions); were over 18; were able to converse in English and had the mental capacity to consent. Participants were given written information regarding the research, and written informed consent was obtained from all participants.

To achieve some diversity, care-givers were recruited via two different routes. The first was the use of a poster displayed in the offices of three different ‘Carers Centres’ in England for approximately 5 months. Two of the Carers Centres displayed the poster in their monthly newsletter. Four participants contacted the researcher after seeing the poster. The second route was attendance by the researcher at three monthly carers’ meetings specifically for mental health care-givers. Information was shared about the research study, and care-givers were asked to contact the researcher if interested in participating. Two participants contacted the researcher following these meetings.

### Data collection

Interviews took place between January and May 2019, with each participant being interviewed once for a duration of 90–120 min.

The interview was piloted to check the order of the questions and length of the interview and to ensure the interview met the research aims as suggested by Bryman (2016). This was piloted with one participant and was found to be relevant to the aims and topic. The researcher decided to remove the question ‘what is your biggest failure in life’ due to the highly emotive connotations. It was also decided the interview would end with a positive question, such as ‘what is your biggest success in life?’. The pilot interview was used in the thematic analysis and compared with the other interviews.

### Data analysis

Each of the six interviews was transcribed by the researcher. The researcher followed Braun and Clark’s (2006) six-stage approach to thematic analysis whilst striving for trustworthiness and credibility, as required by all qualitative researchers (Cope, 2014). This was achieved in the current research by following Guba and Lincoln’s (1994) criteria of being dependable, conforming, credible, transferable and authentic.

The first stage of analysis was to transcribe the data and make a note of any initial ideas and possible patterns that may emerge (Braun and Clarke, 2006). The second stage involved going through each transcript systematically, whilst analysing it line by line and assigning relevant codes. Stage three involved the codes being placed on an excel spreadsheet to search for possible themes. Extracts of the data were included in this process to check the interpretation was true to what the participant said, as required for credibility. Recordings were listened to again, and a reflective diary was utilised to ensure authenticity. Stage four comprised refining and reviewing the themes drawing out a thematic map. Stage five involved giving each theme clear definitions and names. Stages four and five were discussed in supervision to ensure data represent the participants’ response and not the researchers’ values or biases, as required for conformability. The sixth stage involved selecting vivid and compelling extracts and relating the extracts to the research question and previous literature. This ensured dependability and transferability, as the researcher compared the findings to previous research.

### Findings

Three main themes were identified in the data analysis: (1) looking after myself, (2) roles and responsibilities associated with care-giving and (3) service access (see Table 1 for demographic information).

**Table 1.** Participant information.

Pseudonym for care-giver	Pseudonym for care-recipient	Relation: Care-giver–recipient	Care-recipients’ diagnosis	Care-givers’ age	Care-givers’ employment status
Peggy	Mel	Mother–daughter	Schizophrenia	76	Retired
Jasmin	Neema	Step-mother–step-daughter	Schizoaffective disorder	55	Unemployed
Gina	Daniel	Mother–son	Schizophrenia	61	Part-time employment
Jimmy	Sean	Father–son	Paranoid schizophrenia	81	Retired
Mina	Amin	Mother–son	Bipolar	64	Unemployed
Rachael	Simon	Mother–son	Schizophrenia	64	Full-time employment

### Looking after myself

Care-givers shared information about what was important to them, how their hobbies and leisure contributed to their sense of identity and how they engaged in self-care. This involved separating some aspects of their lives from their care-giving role.

Hobbies and leisure activities acted as a coping mechanism allowing care-givers to express themselves as an individual, Jasmin provided an example of this:

I do think my crafty things, going to classes where you are doing something with your hands and colour and you can just switch off and have a laugh with other people and I do think creative outlet is really therapeutic, it’s definitely.

Gina described her relationship with hobbies and leisure activities as being a journey that had developed since initially becoming a care-giver. She recognised this was particularly important due to the non-reciprocal nature of care-giving:

I almost used to feel guilty when one day a week or one night a week I would go out with my friends and go and do an activity. I always used to feel maybe I should put that time for Harry . . . I have come to realise, no, I really need to look after myself as well . . . You have to have a balance basically, but it is all just him, him, him, because he was just taking and he was demanding it

Most care-givers mentioned self-care, their understanding about the importance of looking after themselves had evolved as they gained experience as a care-giver. Self-care was identified as a coping strategy to manage care-giving responsibilities. Mina discussed using mindfulness as a form of self-care:

Drinking a coffee, sitting outside, you know watching TV, I try to do this, I don't say this is but you can’t do anything about it in the past, enjoy the moment

Jimmy identified his ongoing quest to make sense of his life, which for him was his leisure and self-care time:

I’m reading, I’m trying to understand my own life, what happened to my own life

By understanding daily routine and choices before and after becoming a care-giver, it is apparent routines and occupational choices changed and the care-givers adapted as the role progressed. Care-givers valued individuality; less value was placed on co-occupations. Maintaining independent occupations, goals and achievements was important to care-givers and support maintaining occupational competence, which in turn offers a positive occupational identity.

### Roles and responsibilities associated with care-giving

This theme considers how the role of being a care-giver offered new roles and responsibilities which can impact on the ability to engage in other meaningful roles. For parent care-givers, the role is inherited with little choice or control over the matter, and this can influence how the care-giver feels about the role. The role of being a parent to an adult-child with a mental illness impacts not just on the parental relationship but also many other aspects of the individual’s life. Peggy discussed a change in role when her daughter became unwell:

Mel’s illness is probably the biggest thing that happened in my life, the biggest change, I stopped being a mother and started being a carer and that’s where you realised, how awful, how dreadful it was.

Care-givers felt they lacked choice in their care-giving role, due to a sense of obligation, as discussed by Jasmin:

I just sort of fell into it really, I don't really have a choice, that's the other thing because you can't just say 'oh I'm not helping'. . . this situation that I don't want to be in, no one would really want to be in it.

The role of care-giving was found to have a significant impact on other occupations.

All six participants felt their role as a care-giver had impacted on their employment. The impact varied for each individual, with a range of positive and negative experiences. Three of care-givers interviewed ceased working due to their caring responsibilities. Mina felt unable to continue working when her son was diagnosed due to concerns about her son’s behaviour and appearance:

I worked as a Maths teacher being a tutor and many things, but because of Amin I do not want any child coming to my house or to see him or in contact or anything like that

Care-givers experienced concerns about stigma; Gina did not confide in friends or work colleagues due to this:

a lot of people are very ignorant about mental health and it’s not something you want to tell everyone, and I didn't tell my work either

Those who received support and adjustments with their work role were happier in their job, as Rachael shared:

I have my phone on my desk, you are not allowed your phone on the desk when you’re working but I’ve got my phone on silent on my desk just in case I get a call. Because I’ve told them my situation and they know . . . I am very lucky I’ve got a decent job, I’ve got the support of my job

Findings highlighted how the role of being a care-giver altered other roles and relationships within the individual’s life; some gave up meaningful occupations in order to fulfil their care-giving responsibilities.

The non-reciprocal nature of the relationship can make this role difficult. Care-givers found social approval was difficult to attain due to stigma associated with mental illness; some felt unable to seek support from friends and/or colleagues due to stigma. The additional responsibilities care-giving brings to individual’s lives means routines and other meaningful occupations such as productive roles, hobbies and leisure can be interrupted or even ceased. This threatens occupational competence, and care-givers could no longer participate in occupations reflecting their identity.

### Service access

A lack of support from mental health services was overwhelmingly discussed by each care-giver interviewed; there were a total of 171 mentions of unsatisfactory service compared to just 37 mentions of satisfactory or good services by the six participants.

The support received by the care-recipient from mental health services had a direct impact on care-givers and their ability to carry out their responsibilities. Peggy discussed how lack of support in the community can impact on both the care-recipient and care-giver:

They think, by putting somebody in a flat, sending someone out daily to check if they have had their medication, they think that will do. It won't the isolation means people finish back up in psychiatric units . . . Umm, so, as I say from a carers point of view you couldn't give people a better gift than [better community care]

Frustration was felt not only with mental health staff but also the laws and legislation they are required to work within, as Rachael shared:

[the community mental health team] don't do nothing, their hands are tied, what help is he getting? He don't get no help, they are waiting for him to hurt himself or hurt someone and then they can do something. . . And it’s hard because I know it’s his right and I know their hands are tied but it’s not their son

All six participants expressed concerns that the care-recipient was not stimulated and lacked occupational participation and were wasting their lives. Several participants felt mental health services were not doing enough to improve participation. Mina expressed frustration at the lack of support when her son was experiencing depressive episodes:

When he is manic and so, everyone looks at him. The social worker came, the community mental health team, the police and everything because they are frightened if anything happen to society, you know, they don’t care about him… When he is depressed, nobody cares

Care-givers overwhelmingly complained of lack of communication between services, with Gina blaming lack of resources for this:

If they talked to each other and the number of times I’ve had to go through the same thing explaining things to the community care co-ordinator and then to the hospital staff and then to the social worker . . . so you have all these different services they are underfunded, under resourced but they are working hard

Jimmy shared how activities supporting community integration for the care-recipient were valued by care-givers:

these meetings . . .marvellous sort of ideas, meetings in pubs and what not, they seem to really be moving on this idea of promoting a community

Care-givers believed their occupational choices were affected by the lack of support care-recipients received from mental health services. Services play an important role in supporting both the care-recipient and the care-giver although care-givers overwhelmingly experienced a lack of support for the care-recipient which negatively impacted the care-givers’ capability. Care-givers struggled to maintain a satisfying routine which left them feeling their role was unproductive.

When support was offered, this allowed the care-giver more time and choice to engage in other occupations. Care-givers felt the services offered by their local care-givers charity were beneficial, but they did express concerns regarding cuts to these services. Care-givers found law and legislation within the UK both a help and a hindrance. Collaborative services working with the service provider, care-recipient and care-giver were seen as beneficial.

## Discussion

The current research found care-givers were required to change or cease their career, hobbies and routines in order to meet the demands of care-giving which was in line with previous research (Ae-Ngibise et al., 2015; Chaffey and Fosey, 2004; Gater et al., 2014). Occupational adaptation was used as a positive coping strategy by the care-givers interviewed. Weimand et al. (2013) recognised by adapting their values, placing higher value on their own occupational participation and achieving better occupational balance, care-givers were better equipped to cope with the situation although some care-givers changed their occupational roles to be more available for the care-recipient (Chaffey and Fossey, 2004; Weimand et al., 2013).

At times care-givers were found to stretch themselves beyond their limit to engage in their caring role, which was detrimental to coping (Weimand et al., 2013). Participants in the current research also experienced this struggle, giving disproportionate time to their care-giving role although over time their values changed and they recognised the importance of occupational balance. Despite a change in values, care-givers continued to experience a lack of choice which threatens occupational identity (Phelan and Kinsella, 2009). Occupational therapists could use their knowledge of occupational adaptation and occupational identity to support care-givers with this process, which in turn would support them to cope with their new found role.

Care-givers in the current research offered insight into the benefits of maintaining occupations independent of their care-giving responsibilities. Engagement in hobbies and leisure activities offered a welcome distraction for some, allowing them to express themselves as an individual. Occupational participation independent of their care-giving role could be viewed as a coping mechanism, as care-givers recognised their well-being had improved since allowing themselves more time to engage in these occupations. This is supportive of their occupational identity, as they maintained individuality which is at the core of identity formation (Phelan and Kinsella, 2009) although the research was completed in a Western context and might not necessarily be the case for those from Eastern cultures due to more family-orientated identity.

Productivity was a notion valued by all care-givers interviewed in the current research, with some having goals for the care-recipient’s productivity as well as their own. However, they expressed frustration at not being able to meet these goals due to a lack of social and professional support. The role of care-giving had negatively impacted on productivity for all of the care-givers at some point during their care-giving experience which is a threat to their occupational identity (Phelan and Kinsella, 2009). Expectations around productivity and how individuals identify are highly influenced by societal values (Phelan and Kinsella, 2009). If society approves of occupational choices, then individuals are more likely to have a positive occupational identity (Christiansen, 1999).

Mental health care-givers in the current research were found to experience reduced societal approval due to increased stigma associated with socially unacceptable behaviour of the care-recipient or negative societal attitudes. This is a major threat to occupational identity (Phelan and Kinsella, 2009). Comparable results were found in the literature reviewed (Acero et al., 2017; Marimbe et al., 2016; Weimand et al., 2013; Weiss et al., 2013). Fox (2009) named this stigma by association, which leads to care-givers feeling undervalued.

## Implications

The current research has found maintaining occupational participation independent of their care-giving responsibilities supports care-givers to cope with their role. Upon becoming a care-giver, there is inevitably a degree of occupational adaption, which combined with the psychological trauma of having a loved one diagnosed with a chronic mental illness can be very distressing (Acero et al., 2017; Chaffey and Fossey, 2004; McAuliffe et al., 2014). Occupational therapists possess the knowledge and skills to support care-givers in maintaining meaningful occupational participation which in turn provides positive occupational identity and occupational competence.

Participants in the current research and previous research (McAuliffe et al., 2014) suggest mental health professionals could support care-givers by recognising the importance of occupational participation and socialisation for the care-recipient, both elements are vital for care-givers to maintain their role. Care-givers in the current and previous research (Chaffey and Fossey, 2004; McAuliffe et al., 2014) clearly value the skills of occupational therapists as they suggest other mental health professionals do not recognise the importance of occupational participation and socialisation for the care-recipient. Occupational therapists’ holistic approach and unique understanding of the interplay between occupation and health are clearly valued by care-givers and could be utilised to improve care-givers’ well-being. In Australia, occupational therapists developed a peer support programme for mental health care-givers (Bourke et al., 2015) and similar principles could be applied in the UK.

Occupational therapists could utilise the OPHI-II assessment and other Model of Human Occupation assessment tools to understand how care-giving is impacting on occupational participation and occupational identity. Occupational therapists could then offer intervention using occupational therapy–specific knowledge to promote independence and a sense of identity (Royal College of Occupational Therapists, 2017), concepts both valued by the care-givers interviewed in the research although individual cultural values should be considered here as it seems this notion is Western centric (Acero et al., 2017; Marimbe et al., 2016). Utilising occupational therapy knowledge to support care-givers in maintaining a positive occupational identity could improve psychological well-being (Skorikov and Vondracek, 2011) and therefore allow individuals to continue with their role.

## Limitations

The researcher asked care-givers to engage in a face-to-face semi-structured interview. As the care-giving role offers little time to engage in other occupations, this may have limited ability to participate. The care-givers who did participate all had at least 10 years’ experience, and the care-recipient was either in hospital or settled at home, giving the care-giver more free time. Offering the option of virtual interviews or meeting the care-giver at their home may have made the research more accessible to other care-givers and therefore offered different perspectives.

Care-givers were recruited from three different ‘Carers Centres’. This required individuals to identify as a care-giver and have taken the time to register. Only interviewing care-givers who are aware of the services available to them is likely to have caused biases. Recruiting through National Health Service services would have provided an alternative perspective with potential to recruit ‘hidden carers’, who may not have identified as care-givers and had differing experiences.

Given the small number of participants, it was not possible to determine whether saturation of data was reached. A potential bias in the current research is all participants were parents or step-parents, so the findings may not represent other family carers. An additional bias may be five out of the six carers were female. Previous researchers recognised a difference in coping strategies between male and female care-givers (Lawn and McMahon, 2014).

## Further research

The current researcher was unable to use the quantitative measure of the OPHI-II due to small participant numbers and time and resource constraints. Further research could use this to gain a quantitative measure of occupational identity. This could be used to compare with a quantitative measure of stress associated with caring such as the Zarit Burden Interview (Whalen and Buchholz, 2009). This would offer a valid and reliable measure of the relationship between occupational identity and psychological well-being.

## Conclusion

The current research aimed to contribute towards the qualitative evidence base on the occupational identity of mental health care-givers. Based on the literature review, this appears to be the first research project to explicitly examine the occupational identity and occupational experiences of mental health care-givers in the UK. This research has offered further information about how mental health care-givers can be supported by services to maintain their role. Findings from both the current research and literature reviewed substantiated the idea that care-giving for an individual with mental illness can threaten an individual’s occupational participation, negatively impacting on occupational identity. As care-givers gained more experience in their role, they used occupational adaptation as a positive coping mechanism, and this was supportive of their occupational identity.

Occupational therapists already work with individuals with mental illness by supporting them to establish regular routines and engage in meaningful activity which should allow care-givers more time to pursue their own goals. Occupational therapists are well placed to utilise their knowledge of occupational participation and occupational identity to offer advice and guidance to care-givers as well as the care-recipient.

## Key findings

1. Care-giving has a detrimental impact on other meaningful roles. Occupational participation is altered due to the care-giving role which in turn impacts on occupational identity.
2. Maintaining occupations independent of care-giving responsibilities supported individuals to cope.
3. Occupational therapists’ skills and knowledge could support care-givers to cope with their role; they can offer advice and guidance based on their knowledge of occupational participation and occupational identity.
